# Participants’ satisfaction with social security is closely associated with their acceptance of vulnerable groups: a nationwide cross-sectional study in China

**DOI:** 10.3389/fpsyg.2024.1453075

**Published:** 2025-01-08

**Authors:** Chaowei Guo, Yifan Wu, Lina Ge, Li Qi, Yi Ma, Shuang Zang

**Affiliations:** ^1^Department of Community Nursing, School of Nursing, China Medical University, Shenyang, China; ^2^Department of Basic Nursing, School of Nursing, Jilin University, Changchun, China; ^3^Department of Obstetrics and Gynecology, Shengjing Hospital of China Medical University, Shenyang, China; ^4^School of Nursing, Qiqihar Medical University, Qiqihar, China; ^5^Department of Otolaryngology, The First Affiliated Hospital of China Medical University, Shenyang, China

**Keywords:** social security, vulnerable groups, social inclusion, satisfaction, China

## Abstract

**Introduction:**

Social security, as a core component of the national welfare system, has consistently played a crucial role in ensuring the basic livelihood of citizens and promoting social equity and justice. Against this backdrop, this study explores the association between social security satisfaction and acceptance of vulnerable groups.

**Methods:**

This study involved 9923 participants. Generalized linear regression and smooth curve fitting were used to assess the association between social security satisfaction and acceptance of vulnerable groups. Threshold effect was examined by piecewise regression. We conducted subgroup analyses and assessed the potential interaction effect.

**Results:**

A non‑linear association was detected between social security satisfaction and inclusion of vulnerable groups with an inflection point of 45.00. When social security satisfaction was < 45.00, inclusion of vulnerable groups increased with increasing social security satisfaction score up to inflection point. The association between social security satisfaction and inclusion of vulnerable groups differed across gender, education level, and spouse subgroups.

**Discussion:**

The study reveals the importance of social security satisfaction on their acceptance of vulnerable groups. It has a significant meaning in enhancing individuals’ acceptance of vulnerable groups level.

## 1 Introduction

Social exclusion is a universal phenomenon associated with individuals and society (Rawat et al., 2022). When one part of the population is at the center of society, another part is marginalized, and if the marginalized group is unable to successfully reach the center through their efforts, then vulnerable groups are formed (Fu, 2021). The vulnerability of vulnerable groups is reflected not only in economic poverty, but also in social discrimination and violation of legal rights (Limantė and Теrеškinas, 2022; Rani, 2021). The vulnerable groups theory holds that if the vulnerable are not provided with the necessary social protection, they will be more hard to be included by mainstream society and may even be abandoned (Goodin, 1985; Pîrvu and Iordache, 2020). Previous research indicated that people’s attitudes toward vulnerable groups, such as patients with acquired immunodeficiency syndrome, may be influenced by people’s perceived eligibility for social security benefits (Santos et al., 2018; Ulanja et al., 2019). A study in Canada showed that many exonerees struggle to find housing when they are released (Hamovitch et al., 2023). While discrimination based on stigmatization is one of the reasons for this hardship, the deeper reason is that the unavailability of social security for them implies a precarious economic situation (Hamovitch et al., 2022; Cook et al., 2014).

Social security, as a policy lever to provide protection for individuals, plays a prominent role in improving people’s livelihoods (Ullah and Harrigan, 2022). And it is also recognized as a major tool for promoting social inclusion (Catherine and Benstead, 2021; Machin, 2020). It has been shown that increasing social security can increase family acceptance of infertility (Patel et al., 2018, Wang et al., 2021). A research on unemployed people showed that improving the efficiency of the social security system and providing employment for the young unemployed are effective ways to increase society’s willingness to accept them (Yip et al., 2020). Specifically, the higher the level of social security, the more likely participants were to have a positive attitude toward disadvantaged individuals (Yin, 2015; Wang et al., 2021).

A high level of social security means a high satisfaction. Studies have confirmed that satisfactory social security ensures a balanced distribution of national benefits, which is particularly beneficial to enhancing entire vulnerable groups and reducing the gap between individuals (Li and He, 2022; Tri et al., 2021). In addition, social security demonstrates the social fairness and justice, allowing all people to reap a sense of security that their basic livelihoods are guaranteed (Li and He, 2022; Xin, 2021). In other words, the social inclusion of vulnerable groups will be promoted when the public generally obtains the material and spiritual protection provided by social security (Meenakshi and Lalit, 2020).

Therefore, the Chinese government has been committed to investing in strengthening social security in order to improve people’s well-being and satisfaction. Currently, the main form of social security in China is the social insurance system, which provides basic livelihood protection for all citizens (John et al., 2013). Besides, China’s social security also consists of a range of developmental social support or social assistance, such as delivering free medication for people with acquired immunodeficiency syndrome (Ma et al., 2021), providing shelter for homeless individuals (Piao, 2020), and distributing condoms to gender workers (Aimin, 2016). These policies serve as auxiliary measures for vulnerable groups. Under such circumstances, all people can feel the warmth from the country’s overall social security.

To sum up, the association between people’s satisfaction with social security and their acceptance of vulnerable groups deserves to be examined. Previous studies have described the association between satisfaction with social security and participants’ willingness to accept people with criminal records (Nwefoh et al., 2020), infertile people (Shirazi and Rosinger, 2021), and disabled (da Coelho et al., 2020). However, these findings are primarily based on sporadic evidence and limited to select populations and particular regions, and no national study to date has examined the association between satisfaction with social security and the acceptance of all vulnerable groups in China. In this study, we used nationally representative data to analyze the association between participants’ satisfaction with social security and their willingness to accept vulnerable groups, with particular attention to participants’ attitudes to beggars, homosexuals, people released from prison, people of different religions, people with acquired immunodeficiency syndrome, and people cohabitant before marriage. We hope that this study will provide fresh ideas and valuable insights for policy makers and practitioners to better support vulnerable groups.

## 2 Method

### 2.1 Data source

**Figure 1:**
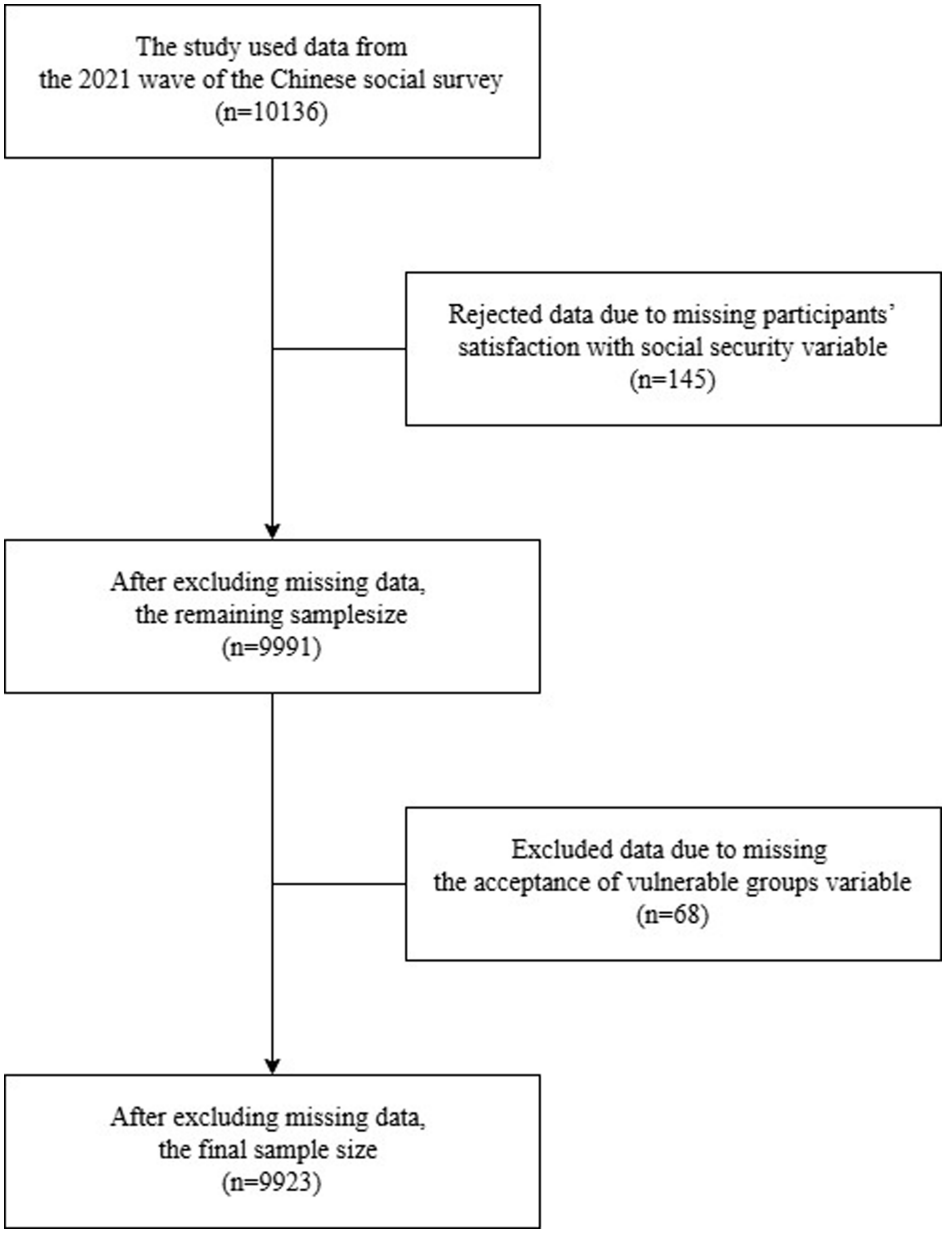
The flowchart of participants included in the present study.

The study used data from the 2021 wave of the Chinese Social Survey (CSS). CSS is a longitudinal study that started in 2006 with follow-up surveys every 2 years. The CSS adopts multi-stage composed sampling. The study is conducted in 31 provinces, autonomous regions, and municipalities directly under the central government, involving 151 counties (districts) and 604 villages (communities), covering over 10,000 households of China’s population. In the first stage of the selection process, counties, cities, and districts were identified as primary sampling units through probability proportionate to size sampling and stratified sampling. In the second stage, the village and committee were sampled through probability proportionate to size sampling. In the third stage, households were randomly selected using address-based random sampling. In the fourth stage, residents were selected by simple random sampling within each household. Details of this survey and its methodology have been published elsewhere (Zhang and Gao, 2021). The survey was conducted through door-to-door visits and face-to-face interviews by interviewers using a standardized structured questionnaire. Interviewers used computer assistants to manage data. The total number of participants in the 2021 CSS was 10,136. Participants who did not answer the questions on social security satisfaction and acceptance of vulnerable groups were excluded. After excluding those with missing values or incomplete answers, the final sample contained 9,923 participants. A selection flow chart can be seen in Figure 1. Ethics approval for this study was granted by the Research Ethics Committee. Informed consent was obtained for all the participants. The data source for CSS was open-access and was available at https://csqr.cass.cn/.

### 2.2 Outcome measures

The acceptance of vulnerable groups was derived from the response to the question “Are you willing to embrace the following people?.” The “people” included beggars, homosexuals, people released from prison, people of different religions, people with acquired immunodeficiency syndrome, and people cohabitant before marriage. The respondents’ reply was assigned as: 4 = very receptive, 3 = more receptive, 2 = less receptive,1 = very unreceptive. People who responded “prefer not to answer” were coded as missing value. The total score was between 6 and 24, a higher score signifies a higher acceptance of vulnerable groups. It has been widely recognized that such an evaluation method could represent the social inclusion of vulnerable groups in China (Dong and Jing, 2021; Li, 2018).

### 2.3 Social security

The independent variable was the participants’ satisfaction with social security. Social security was evaluated by six dimensions: pension security, medical security, employment security, minimum living security, housing security, and comprehensive social security situation. Each item is rated on a 10-point scale, scoring from 1 (very dissatisfied) to 10 (very satisfied). People who responded “prefer not to answer” were coded as missing value. Total summed scale scores ranged from 6 to 60. The higher the score, the higher the participants’ satisfaction with social security. This instrument has been shown to have good assessment validity (Jiang, 2022). The Cronbach’s alpha coefficient obtained in this study was 0.926.

### 2.4 Covariates

Covariates were chosen by examining choice covariates according to the literature (Yang et al., 2020). Age was included in the covariates as a continuous variable. Gender (male vs. female), political status (Communist Party of China member vs. non-party member), education level (primary education and below, secondary education, college education or above), and spouse (without a spouse vs. with a spouse) were included as categorical variables.

### 2.5 Statistical analysis

We checked the data distribution using the Kolmogorov–Smirnov test. The Q–Q plots revealed that the continuous variables age, the participants’ satisfaction with social security, and the acceptance of vulnerable groups did not exhibit a normal distribution. Continuous variables were expressed as median (interquartile range) and categorical variables were expressed as number (percentage). The generalized linear regression was applied in three models to estimate the association between participants’ satisfaction with social security and their acceptance of vulnerable groups. The unadjusted model was not adjusted for covariates, Model I was adjusted for age and gender, and Model II was adjusted for age, gender, political status, education level, and spouse. We used smooth curve fitting to examine whether the independent variable was partitioned into intervals. Segmented regression used a separate line segment that was then applied to fit each interval. *P*-value and β (95% CI) for the non-linearity of the smooth curve fitting were calculated by performing log likelihood ratio tests comparing the non-segmented model to the segmented model. The threshold level of the satisfaction with social security score was determined once the inflection point provided the maximum model likelihood. Additionally, to study comparability, we drew forest plots for subgroup analysis according to the categorical variables in the study (i.e., gender, political status, education level, and spouse). The statistical analyses in this study were performed in R^1^. A *P*-value of less than 0.05 was considered statistically significant.

### 2.6 Sensitivity analysis

We explored the potential for unmeasured confounding between participants’ satisfaction with social security and the acceptance of vulnerable groups by calculating *E*-values (Haneuse et al., 2019). The *E*-value quantifies the required magnitude of an unmeasured confounder that could negate the observed association.

## 3 Results

### 3.1 Characteristics of the study participants

There were a total of 5,524 females (55.67%) and 4,399 males (44.33%) were involved. The age of the participants was 49.00 (35.00–58.00). Most participants were non-partisan (89.78%). More than two-thirds of the participants had received secondary and college education (50.54 and 20.54%, respectively). The participants with a spouse accounted for 78.62%. The full descriptive statistics of study participants are presented in Table 1.

**Table 1:** Characteristics of participants.

Characteristics	Description
Total sample (n)	9,923
Age, median (IQR)	49.00 (35.00–58.00)
Gender, *n* (%)	
Male	4,399 (44.33)
Female	5,524 (55.67)
Political status, *n* (%)	
Communist Party of China member	1,014 (10.22)
Non-party member	8,908 (89.78)
Missing value data	1 (0.01)
Education level, n (%)	
Primary education and below	2,861 (28.83)
Secondary education	5,015 (50.54)
College education or above	2038 (20.54)
Missing value data	9 (0.09)
Spouse, *n* (%)	
Without a spouse	2,118 (21.34)
With a spouse	7,801 (78.62)
Missing value data	4 (0.04)
Social security, median (IQR)	36.00 (24.00–48.00)
The acceptance of vulnerable groups, median (IQR)	13.00 (11.00–16.00)

The sum does not equal 100% owing to rounding. IQR, interquartile range.

### 3.2 Associations between participants’ satisfaction with social security and their acceptance of vulnerable groups

We analyzed the associations between participants’ satisfaction with social security and their acceptance of vulnerable groups using generalized linear regression in three models. In the unadjusted model, there was a significant association between participants’ satisfaction with social security and their acceptance of vulnerable groups (*β* = 0.054, 95% CI = [0.049, 0.059], *P* < 0.001). After adjusting for potential confounders, the associations between participants’ satisfaction with social security and their acceptance of vulnerable groups were still robust in Model I and Model II (Table 2). As for Model I and Model II, at each point that satisfaction with social security increases, the acceptance of vulnerable groups increases by 3.70 and 3.00%, respectively.

**Table 2:** Association between participants’ satisfaction with social security and their acceptance of vulnerable groups (*n* = 9,923).

Outcome	Unadjusted model		Model I		Model II	
	*β* (95% CI)	*P*-value	*β* (95% CI)	*P*-value	*β* (95% CI)	*P*-value
Acceptance	0.054 (0.049,0.059)	<0.001	0.037 (0.032,0.041)	<0.001	0.030 (0.025,0.034)	<0.001

Adjustments: Model I: age and gender; Model II: age, gender, politics status, education level, and spouse.

### 3.3 Association of participants’ satisfaction with social security and their acceptance of vulnerable groups

**Figure 2:**
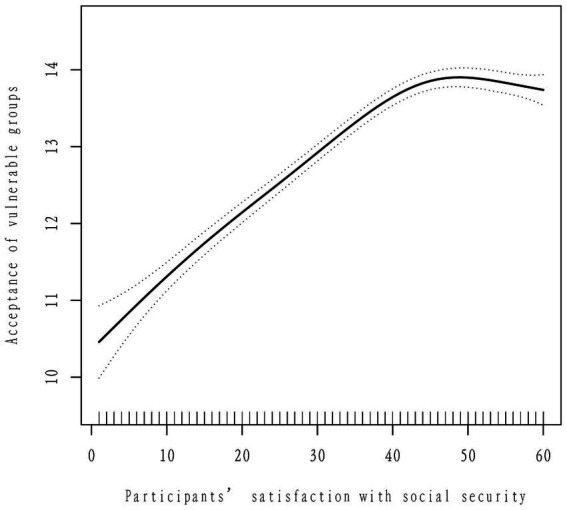
Smoothing analysis curve for the acceptance of vulnerable groups and the satisfaction with social security. The black continual line is a fitting line of participants’ satisfaction with social security and their acceptance of vulnerable groups, and the interval between the black dot lines is 95% confidence interval.

As shown in the smoothing plots, there was a non-linear association between participants’ satisfaction with social security and their acceptance of vulnerable groups, showing an inverted J-shaped. Then, the segmented model (piecewise regression model) was used to evaluate the threshold effect of the fitted curve. The log-likelihood ratio test of satisfaction with social security at the inflection point was statistically significant (*P* < 0.001), suggesting that the segmented model was appropriate for describing the association between participants’ satisfaction with social security and their acceptance of vulnerable groups. The inflection point for the satisfaction with social security score was 45.00 (Figure 2).

When participants’ satisfaction with social security score < 45.00, their acceptance of vulnerable groups increased significantly with the increasing satisfaction with social security (*P* < 0.001). And the acceptance of vulnerable groups increased by an average of 7.80% with each point increase in social security satisfaction (*β* = 0.078, 95% CI = [0.071, 0.085]). When participants’ satisfaction with social security score ≥ 45.00, their acceptance of vulnerable groups decreased significantly with the increasing satisfaction with social security (*P* = 0.005). And the acceptance of vulnerable groups decreased by an average of 2.50% with each point increase in social security satisfaction (*β* = −0.025, 95% CI = [−0.043, −0.008]) (Table 3).

**Table 3:** Threshold effect analysis of participants’ satisfaction with social security and their acceptance of vulnerable groups (*K* = 45.00).

Value	Non-segmented model	Segmented model	
		Social security < K	Social security ≥ K
*β* (95% CI)	0.054 (0.049, 0.059)	0.078 (0.071, 0.085)	−0.025 (−0.043, −0.008)
*P*-value	<0.001	<0.001	0.005

### 3.4 Subgroup analysis

**Figure 3:**
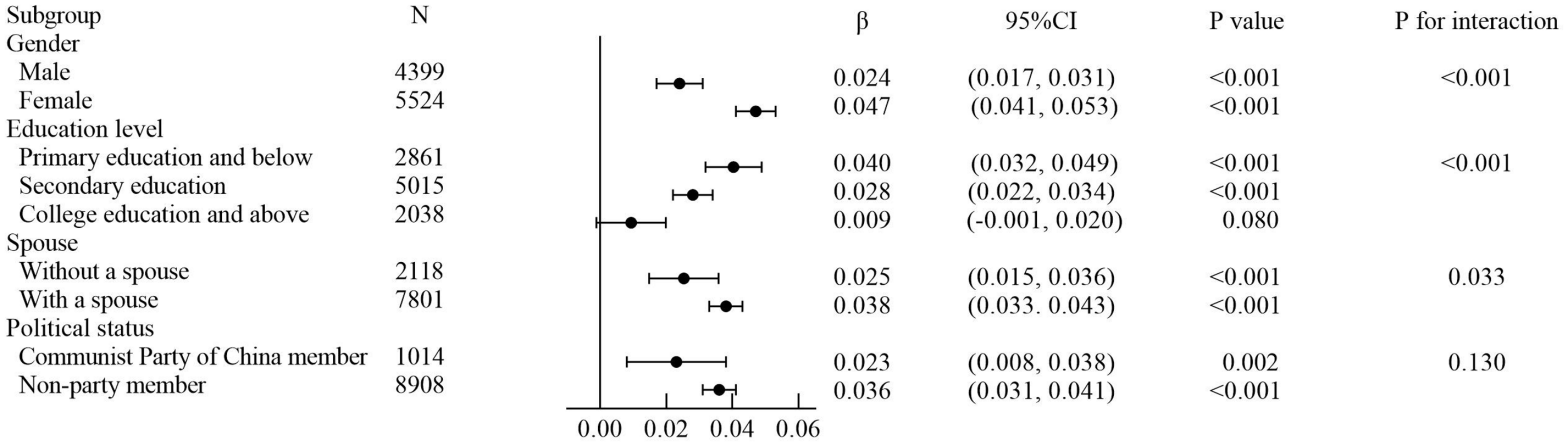
Association between the satisfaction with social security and their acceptance of vulnerable groups in subgroups. Stratified by gender, political status, education level, and spouse. The forest plot of the subgroup analysis was adjusted for age. Line bars indicate 95% CI. CI, confidence interval.

We conducted subgroup analyses by four predefined factors (gender, political status, education level, and spouse) (Figure 3). The positive association between participants’ satisfaction with social security and their acceptance of vulnerable groups was generally persistent across these subgroups. Significant interactions of satisfaction with social security with gender (male vs. female, *β* = 0.024 [0.017, 0.031] vs. *β* = 0.047 [0.041, 0.053]; *P* for interaction <0.001), educational level (primary education and below vs. secondary education vs. college education and above, *β* = 0.040 [0.032, 0.049] vs. *β* = 0.028 [0.022, 0.034] vs. *β* = 0.009 [−0.001, 0.020]; *P* for interaction <0.001), and spouse (without a spouse vs. with a spouse, *β* = 0.025 [0.015, 0.036] vs. *β* = 0.038 [0.033, 0.043]; *P* for interaction = 0.033) were observed. There were significant differences in the association of satisfaction with social security with the acceptance of vulnerable groups in gender subgroup, education level subgroup, spouse subgroup, indicating that there was an interaction effect (*P* for interaction <0.05). The associations were more pronounced in female than in male. The associations were more pronounced among those with primary education and below than among those with secondary education. The associations were more pronounced among those with a spouse than among those without a spouse. The association of satisfaction with social security with the acceptance of vulnerable groups was not statistically different within different political status groups, indicating that there was no interaction effect (*P* for interaction >0.05). The political status had no effect on the association between social security satisfaction and acceptance of vulnerable groups.

### 3.5 Sensitivity analysis

The *E*-value of this study was 4.34. Based on the *E*-value, it is unlikely that unmeasured confounding factors could fully explain our results.

## 4 Discussion

In this nationwide cross-sectional study, we found an association between participants’ satisfaction with social security and their acceptance of vulnerable groups. Previous studies have suggested that when there is inadequate social security or if the evaluation of the social security system is not highly rated, participants are reluctant to accept vulnerable groups in society, such as immigrants (Aguila and Vega, 2017). When the satisfaction with social security decreases, social contradictions will increase (Zhang and Liu, 2019), which is not conducive to residents’ acceptance of vulnerable groups. Additionally, the increase in participants’ satisfaction of social security increases their willingness to accept the vagabond, which promotes social integration (Zheng, 2005). A previous study also indicated that sound social security services play an important role in increasing the sense of gain and happiness of the public who handle social insurance services and according to promote their acceptance of vulnerable groups (Wen and Mao, 2021). When participants’ social security is improved, they will take the initiative to pay attention to vulnerable groups and take the responsibility to accept them. As social security redistributes part of participants’ income, including redistribution both among high and low-income people, it reduces the gap between different people and promotes social integration (Yang and Zhao, 2022).

When the satisfaction with social security score was less than 45.00, the acceptance of vulnerable groups increased as Chinese participants’ satisfaction with social security increased. Participants’ satisfaction with social security score of 45.00 was associated with optimal acceptance of vulnerable groups. This may be explained by the fact that people with high social security satisfaction can easily translate the thought of accepting vulnerable groups into concrete actions, such as reducing discrimination against people with acquired immunodeficiency syndrome (Ren et al., 2021). The transition from beliefs and attitudes to taking practical action often requires sufficient motivation, social resources, and opportunities. As cognitive dissonance theory argues, consistency between beliefs and perceptions facilitates action, whereas inconsistency creates a discomfort that reduces the likelihood of action (Festinger, 1957). Previous research has suggested that clear welfare-consequentialism policies can make participants’ lives better (Noel, 2020). One such factor was the public perception of the reliability of the social security system. People may have more confidence in their lives when they believe that social security is reliable and stable. Therefore, governments should make welfare policies clear to participants. Since 2013, the Chinese government has taken some measures to improve social security, such as putting forward the strategy of targeted poverty alleviation (Chen and Li, 2013; Jiang and Lu, 2022). Meanwhile, a social assistance network has been established to provide support to vulnerable groups, laying the foundation to achieve inclusive development in China. These initiatives were a generalized system of preferences, therefore, the impact of this welfare was felt not only by aid recipients but also by the masses.

The threshold effect of this study showed that when participants’ satisfaction score of social security was above 45.00, the improvement of participants’ satisfaction of social security was associated with a decrease in their willingness to accept vulnerable groups. This may be because these participants feel social security is sufficient, so they may not intend to provide additional support for vulnerable groups (Lee et al., 2018). Additionally, one possible explanation is that individuals who receive more social security benefits may feel they have worked hard for their benefits and regard social security benefits as an entitlement they have earned, rather than as a form of assistance given to those in need (Bi, 2022). Meanwhile, people with more adequate social security are typically higher-income people (François et al., 2020). Higher-income residents are more likely to resist the placement of such facilities in their neighborhoods because they perceive them as potentially lowering property values or disrupting the character of the community (Dear, 1992; Martin and Myers, 2005). It is also possible that there may be a lack of understanding or empathy toward vulnerable populations due to the great disparity in social status (Carter et al., 2020; Ruggs et al., 2023). Social status can create a disconnect between people with adequate social security from the struggles and challenges faced by marginalized individuals and communities (Lu, 2006). This can result in a lack of awareness or concern for the needs of vulnerable groups by those who are already fully satisfied with social security. Finally, people with adequate social security may view vulnerable groups as a personal failing rather than as suffering from systemic societal issues. They may believe that vulnerable groups who are homeless (Li, 2013), unemployed (Lei, 2016), or struggling with addiction (Wang, 2021) are solely responsible for their situations, rather than recognizing the broader societal and economic factors that may contribute to these issues. Therefore, society needs the spirit and vibe related to humanitarian deliberation and values that support vulnerable groups is clear.

The inverted J relationship between social security satisfaction and acceptance of vulnerable groups underscores the complexity of public sentiment toward social welfare. This pattern suggests that there is a threshold at which increased satisfaction leads to a peak in acceptance, after which further increases in satisfaction may lead to complacency or a sense of entitlement. The inverted J relationship between social security satisfaction and acceptance of vulnerable groups is further complicated by the interaction effects of gender, educational level, and civil status. Research by Hauspie et al. (2023), highlights gender differences in response to academic predictors, which may extend to social security perceptions and acceptance. Additionally, tailored interventions, as discussed by Hugh et al. (2023), are crucial for effectively addressing the diverse needs of different demographic groups within social security frameworks.

However, participants’ satisfaction of social security is not only a reflection of the economic situation, but also an emotional outgrowth (Robert, 2004). As a consequence, participants’ satisfaction of social security may not reflect the real social security situation accurately. Furthermore, social security is subject to ongoing changes and reforms, which can further complicate participants’ perceptions of social welfare program. It is therefore necessary to consider promoting social security improvements or provide education and incentives to promote better acceptance of vulnerable groups in society.

Our study found that the association between the satisfaction with social security and willingness to accept vulnerable groups was particularly prominent among females. Previous studies have demonstrated that females are more receptive to certain vulnerable groups, such as people who cohabitant before marriage (Han et al., 2021). Whereas attitudes of males toward vulnerable groups such as lesbians and male homosexuals were negative, especially toward male homosexuals (Yan et al., 2009). One possible explanation is that females have a significantly higher sense of community than men (Chen et al., 2018) and that females treat vulnerable groups usually more tolerant. Moreover, females are better at perspective-taking and empathy than men, and they, therefore, are inclined to understand the situation of vulnerable groups (Wang et al., 2015). Our study also found that the association between the satisfaction with social security and willingness to accept vulnerable groups was particularly prominent in the subgroup with a primary education and below. This may be due to shortcomings in social security, including a shortage of qualified teachers, low salaries, overcrowded public schools, and inadequate facilities and equipment in schools serving vulnerable populations (Nzoka, 2013). China lacks inclusive schools that integrate ordinary populations with vulnerable groups (Cao et al., 2023). Although individuals with higher education and the general public are aware of the existence of inclusive schools, they do not support this form of education, thereby limiting interactions between vulnerable groups and highly educated individuals (Bujorean, 2018; Neufeldt and Mathieson, 1995). Nevertheless, at the compulsory education level, such as free primary education, efforts have advanced societal inclusion for vulnerable populations (Hu and Roberts, 2011).

Another element to highlight is the association between the satisfaction with social security and participants’ willingness to accept vulnerable groups was attenuated among those who had no spouse. Similar to our findings, Yu et al. (2016) found that participants who have support from their spouses feel they can take in the patient with acquired immunodeficiency syndrome. A possible explanation is that getting support from spouses can help participants build a good mentality to face vulnerable groups (Ying et al., 2016). In addition, research shows that participants mainly get emotional support from their spouses (Yuan and Fang, 2016), and emotional support impacts participants’ ability to empathize with vulnerable groups (Liu et al., 2014), which lays the foundation for the participant’s acceptance of vulnerable groups.

The interaction effects of gender, educational level, and marital status refer to the influence of these variables on the association of participants’ satisfaction with social security satisfaction and their acceptance of vulnerable groups. Previous study have found that gender, education level, and marital status are factors that affect social security satisfaction and acceptance of vulnerable groups (Choukou et al., 2022). Gender may influence citizens’ concerns about social security and vulnerable groups (Forbes et al., 2008). For example, female usually pay more attention to issues such as housing security and comprehensive social security situation, so their attention to the acceptance of vulnerable groups may be more inclined to the protection of the rights and interests of vulnerable female and people cohabitant before marriage. While male may pay more attention to employment security or pension security, which may affect the strength of the association between social security satisfaction and the acceptance of vulnerable groups. Gender roles influenced by marital status may also amplify or weaken the association between social security satisfaction and acceptance of vulnerable groups (Riaz et al., 2023). For example, in traditional societies, female with spouses may pay more attention to the social security of the family and at the same time have less contact with vulnerable people due to the constraints of family and social responsibilities, thus lacking a deep understanding of the situation of vulnerable people. Thus, unmarried female may have a higher acceptance of vulnerable groups. Male with a high school education or above and have a spouse may be more concerned with seeking social security for their family through individual efforts and neglect vulnerable groups. Individuals with lower educational levels may be more influenced by traditional values, such as poverty alleviation and helping those in need or neighborly mutual assistance, and thus tend to show a higher level of acceptance toward vulnerable groups. This highlights the importance of considering the complex interplay of demographic variables when examining social security satisfaction and acceptance of vulnerable groups.

### 4.1 Limitations

There were several potential limitations to the study to note. First, the data collection was conducted among Chinese participants, so the responses regarding willingness to accept vulnerable groups may be restricted to cultural background. Second, data were self-reported and therefore might be subject to recall bias. Third, although *E*-value showed robust results in this study, there were still some potential confounding factors that could not be controlled. Fourth, our analysis was based on cross-sectional data, thereby limiting causal inferences about willingness to accept that vulnerable groups may exist.

## 5 Conclusion

Better integration of vulnerable groups into society and acceptance by all contributes to social inclusion. This study found that participants’ satisfaction of social security was closely associated with the acceptance of vulnerable groups. Specifically, there was an inflection point in the association between satisfaction with social security and the acceptance of vulnerable groups. In terms of implications for the future, this suggests that policies aimed at improving social security programs could create a more supportive and inclusive society.

## Data Availability

The original contributions presented in the study are included in the article/supplementary material, further inquiries can be directed to the corresponding authors.

## Footnotes

^1^ http://www.R-project.org
